# Optimal Sub-Band Analysis Based on the Envelope Power Spectrum for Effective Fault Detection in Bearing under Variable, Low Speeds

**DOI:** 10.3390/s18051389

**Published:** 2018-05-01

**Authors:** Hung Ngoc Nguyen, Jaeyoung Kim, Jong-Myon Kim

**Affiliations:** School of Computer Engineering and Information Technology, University of Ulsan, Ulsan 44611, Korea; hungnguyenvldt@gmail.com or hungnguyen85@mail.ulsan.ac.kr (H.N.N.); kjy7097@gmail.com or kim7097@ulsan.ac.kr (J.K.)

**Keywords:** fault detection, sub-band analysis, DWPT, health-related index (HI), envelope power spectrum (EPS), bearing defects

## Abstract

Early identification of failures in rolling element bearings is an important research issue in mechanical systems. In this study, a reliable methodology for bearing fault detection is proposed, which is based on an optimal sub-band selection scheme using the discrete wavelet packet transform (DWPT) and envelope power analysis techniques. A DWPT-based decomposition is first performed to extract the characteristic defect features from the acquired acoustic emission (AE) signals. The envelope power spectrum (EPS) of each sub-band signal is then calculated to detect the characteristic defect frequencies to reveal abnormal symptoms in bearings. The selection of an appropriate sub-band is essential for effective fault diagnosis, as it can reveal intrinsically explicit information about different types of bearing faults. To address this issue, we propose a Gaussian distribution model-based health-related index (HI) that is a powerful quantitative parameter to accurately estimate the severity of bearing defects. The most optimal sub-band for fault detection is determined using two dimensional (2D) visualization analysis. The efficiency of the proposed approach is validated using several experiments in which different defect conditions are identified under variable, and low operational speeds.

## 1. Introduction

Induction motors are the most commonly used rotating machines in industrial applications due to their commercial availability, reliability, and reasonable cost. However, they are often used in extreme working conditions, which can cause unexpected failures leading to unanticipated interruptions in industrial production. Hence, the early and accurate detection and identification of various faults can be helpful in preventing unexpected failures in induction machines. Rolling element bearings account for more than 50% of faults in rotating machinery [1]. Bearings are critical components in the motors of wind turbines, helicopters, gearboxes, and automobiles; they help make the relative movement of elements in systems smoother. Bearing defects are common and often arise due to adverse conditions in the operating environment, corrosion, problems with lubrication, assembly, misalignment, and overloading [1]. Consequently, bearing defects are the primary cause of abrupt mechanical breakdowns in the industry, which can lead to large economic losses or even result in catastrophic consequences [2]. Therefore, the precise detection of incipient faults in rolling element bearings is required for reliable condition monitoring and maintenance of industrial machines.

Advanced data acquisition and processing techniques have been developed for effective and early fault detection and identification in critical systems. In existing approaches, bearing failures have been successfully diagnosed using different signal processing methods to extract characteristic defect features via vibration analysis [3,4,5], current signature analysis of induction motors [6,7,8], and stray flux measurement [9]. These techniques have been extensively applied and have been very effective in detecting and identifying various bearing faults. Moreover, stray fluxes radiating out of the induction motor have been used to detect various defects. The stray flux radiated by an induction motor can be affected by the presence of different faults. The correct measurement and correlation of changes in stray flux with different faults has been used for the identification of different faults in the induction motor. However, vibration analysis and stator current signature analysis are effective for bearing fault diagnosis only at high rotational speeds. These vibration and stator current analysis based techniques are ineffective at capturing defect symptoms at low rotational speeds. To overcome this problem, acoustic emission (AE)-based techniques have been exploited for low-speed bearing fault diagnosis [10,11]. AE signals can be used to capture low-energy fault signatures that are characteristic of incipient defects at low operating speeds [12,13]. This advantage makes AE-based processing methods more attractive and useful in many applications. Hence, a reliable technique is presented in this paper for the diagnosis of incipient bearing defects at low rotational speeds using AE analysis.

Early bearing faults are mainly detected by analyzing the acquired AE signals for abnormal symptoms. When a rolling element passes across a defect on the bearing surface, an impulse is generated. These impulses are generated periodically according to the frequency with which the rolling elements pass over the defect on the bearing surface, exciting higher frequency bearing resonances in the process [14]. These higher frequency bearing resonances act as carrier, and are amplitude modulated by the modulating signal, i.e., the defect signal. The defect signal is the series of periodic impulses generated when rolling elements of the bearing pass over a defect on the bearing surface. The AE sensor captures these amplitude modulated signals. The detection of defect signals and hence the diagnosis of different defects in bearings requires the use of demodulation techniques. Envelope power analysis is the most effective technique for signal demodulation and hence the detection of defect frequencies associated with each type of localized bearing defect [15,16]. These modulated defect frequencies, which can be used to detect incipient bearing defects, can appear in different sub-bands of the envelope power spectrum (EPS). It is very difficult to directly identify these defect frequencies as they typically have low amplitudes and are easily disturbed by other noise components. Therefore, signal-processing approaches, such as short time Fourier transform (STFT) [17], wavelet transform (WT) [18,19], and band-pass filter banks [20], have been explored along with EPS analysis for the detection of these defect frequencies and hence localized defects in bearings. In this paper, discrete wavelet packet transform (DWPT)-based decomposition is first performed on the condition monitoring AE signals for bearings. Then, the EPS is calculated to detect intrinsic information about the bearing faults. The DWPT is better than the discrete wavelet transform (DWT) as it provides uniform resolution in both the high- and low-frequency domains to render a more informative and efficient description of the original signal [21]. However, there is still no general consensus as to which sub-band is optimal for reconstruction and the calculation of the EPS, which can then be used for the diagnosis of different bearing defects. In general, for the EPS obtained from sub-band signals, kurtogram-based methods have been widely used to discover the appropriate sub-band that contains useful information intrinsic to various bearing defects [16,22,23]. However, these techniques are not always effective in identifying the optimal sub-band due to the inclusion of other components, such as the harmonics of the operating frequency and noise frequencies in their spectra. To overcome this problem, we present an advanced approach for assessing the severity of defects in bearings by using a Gaussian distribution model (GDM)-based health-related index (HI). It is computed by calculating the ratio between defective spectral components and initial spectral components in the EPS. The HI is a meaningful and effective metric to estimate the severity of bearing defects. Furthermore, an analysis tool for two dimensional (2D) visualization, which represents the HI value-based fault degrees of sub-bands, is exploited to select the optimal sub-band for bearing fault detection.

This paper proposes a reliable fault detection method for rolling element bearings by combining DWPT decomposition and GDM-based envelope analysis of AE signals. The main contributions of this paper are:

- A DWPT-based sub-band analysis is performed to extract the characteristic features of different bearing faults from the acquired AE signals. The symptoms of incipient bearing defects are detected by calculating the EPS of each sub-band signal at different decomposition levels. Furthermore, a GDM-based HI calculation is presented for quantifying the severity of defects.
- The HI values of fault components are computed by determining the Gaussian windows around the characteristic defect frequencies and their harmonics. A 2D visualization tool, representing the HI values obtained from the EPS, is used to find the most informative sub-band with the highest HI value for fault detection.
- The efficiency of the proposed bearing fault detection approach is validated using different defect conditions under low and variable rotational speeds.

The rest of this paper is organized as follows: Section 2 describes the data acquisition and bearing characteristic frequencies. Section 3 presents the proposed bearing fault detection scheme with optimal sub-band signal analysis via the proposed GDM-based evaluation of defect severity. Section 4 discusses the experimental results. Finally, Section 5 concludes this paper.

## 2. Bearing Fault Data Acquisition System

**Figure 1:**
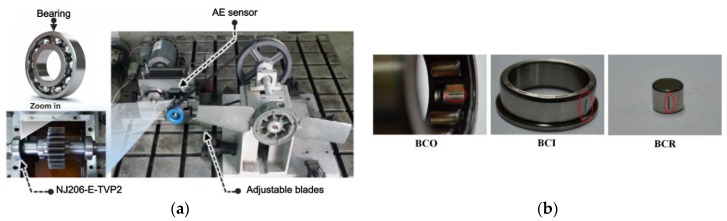
(**a**) AE data acquisition system; and (**b**) Primary defect conditions of bearing.

A machinery fault simulator is set up to study the different defect types in bearings, as illustrated in Figure 1a. The fault simulator has two shafts, i.e., a drive end shaft and a non-drive end shaft, which are connected to each other through a gearbox on the inside. Both ends of the shafts are fastened using FAG NJ206-E-TVP2 (NSK, Seoul, Korea) cylindrical rolling element bearings. In addition, the non-drive end shaft can be loaded using a fan with two adjustable blades. The fan is connected to the shaft via a belt and pulley. The acoustic emission (AE) activity generated by the seeded defects in bearings is captured using an AE sensor, WSα, which is fixed on top of the bearing housing, as shown in Figure 1a. Moreover, this AE sensor is directly connected to a PCI board system with 2-channels for data acquisition to digitize the obtained continuous AE signals for further processing. Table 1 lists the detailed specifications of the AE sensor-based data acquisition system. In this paper, the AE signals related to bearing defects are recorded at a sampling frequency of 250 kHz for a duration of 5 s each. The bearings used in this study are seeded with defects at three different locations on the bearing surface, i.e., a crack on the outer race (BCO), a crack on the inner race (BCI), and a crack on the roller (BCR), as illustrated in Figure 1b. Each time a roller strikes a fault on the bearing surface, and an impulse is generated. Since the rollers rotate at a fixed frequency, these impulses are generated at a frequency that is characteristic to each defect type. These characteristic defect frequencies are the ball pass frequency of the outer race (BPFO), ball pass frequency of the inner race (BPFI), two times the ball spin frequency (2 × BSF), and the fundamental train frequency (FTF) corresponding to the rotational speed of the cage, as defined in [15]. These defect frequencies can be calculated using Equation (1) for a bearing of known geometry and rotating at a constant rotational frequency. The values of these defect frequencies for the bearings used in this study are given in Table 2.

These defect frequencies depend upon the rotational speed of the bearing, the bearing geometry, and fault location, which are defined using the following parameters in Equation (1): the number of balls (n_roller_), shaft speed in Hertz (F_r_), contact angle of the ball and races (α), roller (or ball) diameter (B_d_), and pitch diameter (P_d_). These characteristic defect frequencies can be searched for in the envelope power spectrum of the AE signal for the detection of corresponding defects in bearings. In this study, the AE signals for each defect type are recorded under variable rotating speeds.

(1) $$\begin{array}{l} \mathrm{BPFO}=\frac{n_{\mathrm{roller}}.F_{r}}{2}\left(1-\frac{B_{d}}{P_{d}}\cos\alpha \right)\\ \mathrm{BPFI}=\frac{n_{\mathrm{roller}}.F_{r}}{2}\left(1+\frac{B_{d}}{P_{d}}\cos\alpha \right)\\ \mathrm{BSF}=\frac{P_{d}.F_{r}}{2.B_{d}}\left(1-{\left(\frac{B_{d}}{P_{d}}\cos\alpha \right)}^{2}\right)\\ \mathrm{FTF}=\frac{F_{r}}{2}\left(1-\frac{B_{d}}{P_{d}}\cos\alpha \right) \end{array}$$

## 3. The Proposed Methodology for Bearing Fault Detection

**Figure 2:**
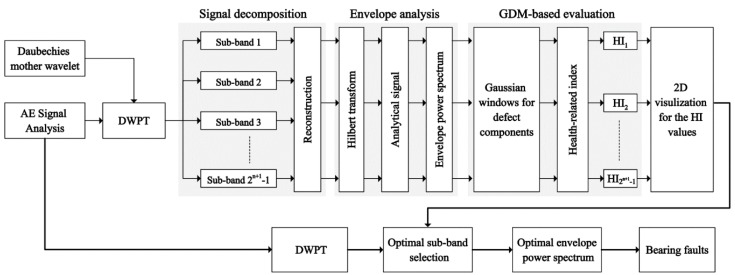
The overall block diagram of a proposed bearing fault detection system.

The proposed methodology for bearing fault detection is described in Figure 2. The AE signal for each bearing defect is decomposed into a set of sub-bands using the DWPT. These sub-bands are then reconstructed using the inverse DWPT, and for each reconstructed signal the envelope power spectrum is constructed. The envelope power spectrum is constructed by first calculating the Hilbert transform of each reconstructed signal, and then using it to calculate the analytic signal. The Fourier transform of the analytic signal is then calculated to get the envelope power spectrum (EPS) of the reconstructed signal. Finally, for the EPS of each reconstructed signal, a Gaussian distribution model (GDM)-based health-related index (HI) is calculated. The optimal sub-band is then determined by the 2D visualization of these HI values. The sub-band with the highest value of HI for its EPS is the optimal sub-band that contains the most information about different bearing defects. Bearing defects are then diagnosed by taking the AE signal, decomposing it into different sub-bands, reconstructing the signal from the optimal sub-band only and then calculating the EPS of this reconstructed signal. The optimal EPS is then used for diagnosing different bearing defects. More details of the proposed method are given below.

### 3.1. Envelope Analysis for DWPT-Based Sub-Band Signals

**Figure 3:**
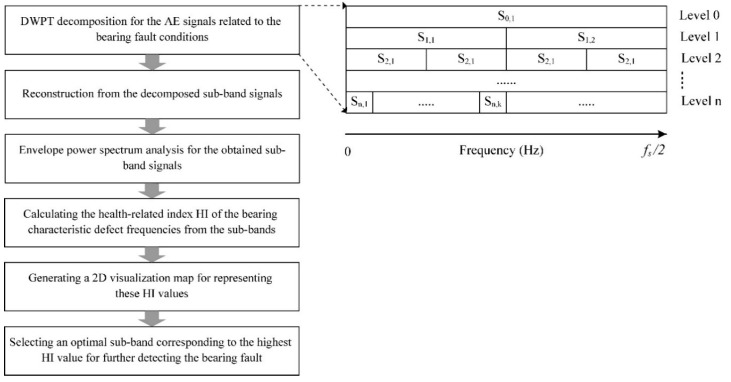
An analysis process of the sub-band signals based on EPS and DWPT decomposition.

The AE signals are analyzed based on the assumption that they contain information about all of the possible bearing defects considered in this paper, including BCO, BCI, and BCR. For the purpose of detecting these fault signatures, envelope analysis is typically used to extract the fault frequency components from the acquired AE signals. However, intrinsic information about the bearing defects can occur anywhere in the frequency analysis domain. Therefore, to correctly identify the bearing fault signatures, envelope analysis is performed on each sub-band signal decomposed by the DWPT. The sub-bands containing the most useful information about the bearing defects are determined via the GDM-based HI calculation. The overall process of DWPT-based envelope analysis is illustrated in Figure 3, where f_s_ is the sampling frequency of the AE signal and S_n,k_ is the k^th^ sub-band signal at the n^th^ level.

First, the DWPT is employed to decompose the 5 s input AE signal into a series of uniform frequency sub-bands with four levels of decomposition. The Daubechies mother wavelet function is frequently utilized as a signal filter due to its effectiveness in analyzing abnormal symptoms of bearing defects [22,24]. The four-level decomposition using DWPT and the Daubechies 4 (db4) mother wavelet function is carried out to obtain 2^5^ − 1 sub-band signals. A reconstruction step is then performed for each sub-band, and the EPS of each reconstructed sub-band signal is calculated using the Hilbert transform [19,25]. The EPS of each sub-band signal will contain the characteristic defect frequencies and their harmonics, if there is a defect in the bearing. The precise capturing of the bearing defect frequencies and their harmonics (BPFO, BPFI, and 2 × BSF) is essential for reliable fault detection. Therefore, in order to assess the EPS of a given sub-band for the presence and strength these characteristic defect frequencies, the proposed GDM-based HI is measured by calculating the ratio between the defective spectral components and the initial spectrum components of the EPS. A detailed description of the steps involved in the calculation of HI is given in Section 3.2. After obtaining the HI values for the envelope power spectrum of each sub-band signal, a 2D visualization tool is used to represent the HI values of all the sub-bands. Through 2D visualization analysis, the highest HI value is used to determine the most informative sub-band for evaluation, as shown in Figure 3. Hence, the optimal sub-band signal that is most suitable for detecting the characteristic defect frequencies associated with outer race, inner race, and roller defects is selected based on the highest HI value.

### 3.2. Gaussian Distribution Model-Based Calculation of Defect Components

**Figure 4:**
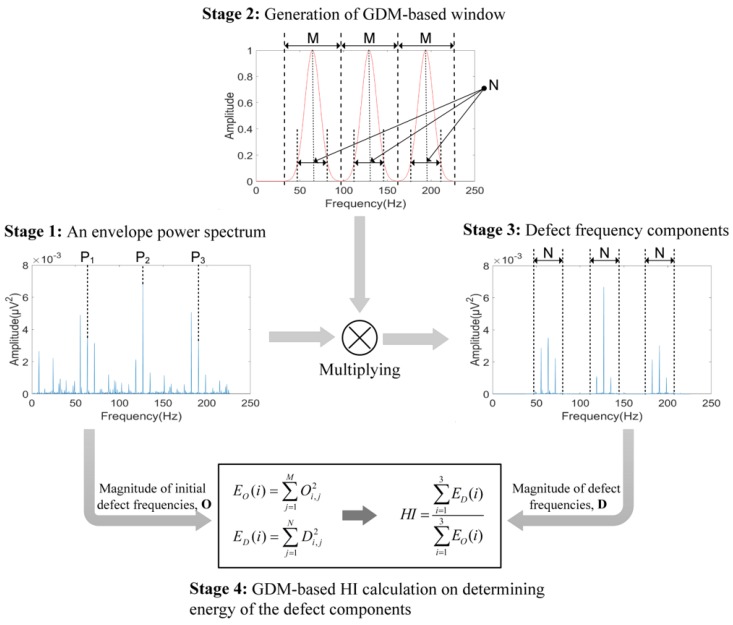
A description of the GDM-based HI calculation stages for each sub-band.

In order to detect the defect frequencies and their harmonics, i.e., BPFO, BPFI, and 2 × BSF, a GDM-based method is proposed for calculating HI of the EPS. The HI is calculated by using Gaussian windows around the harmonics of these defect frequencies, i.e., BPFO, BPFI, and 2 × BSF. The steps involved in the computation of GDM-based HI are illustrated in Figure 4 and described below.

Stage 1: First, the envelope power spectrum (EPS) of a given sub-band signal is calculated. The calculation of EPS first requires the computation of the Hilbert transform of the reconstructed sub-band signal x_sb_(t) [19], which is defined in Equation (2). The Hilbert transform is used to demodulate the original signal and to subsequently construct the envelope signal. The calculation of the envelope signal is necessary for the recovery of the low-frequency defect components from the high frequency carrier signal. The AE signal recorded by the AE signals is a modulated signal, where the low magnitude defect frequencies modulate the high magnitude bearing resonances, which act as carrier frequencies:

(2) $${\hat{x}}_{sb}(t)=\frac{1}{\pi }\int_{-\infty }^{+\infty }\frac{x_{sb}(\tau )}{t-\tau }d\tau$$

The original time-domain sub-band signal x_sb_(t) is combined with the Hilbert transformed signal ${\hat{x}}_{sb}(t)$ to generate the analytical signal s(t):

(3) $$s(t)=x_{sb}(t)+j.{\hat{x}}_{sb}(t),\;\text{with}\;j=\sqrt{-1}$$

The envelope signal $x_{\mathrm{env}}(t)$ is basically the magnitude of the analytic signal s(t), which can be determined as follows:

(4) $$x_{\mathrm{env}}(t)=\left|s(t)\right|=\sqrt{x_{sb}{\left(t\right)}^{2}+{\hat{x}}_{sb}{\left(t\right)}^{2}}$$

Finally, the fast Fourier transform (FFT) of the envelope signal $F\{x_{\mathrm{env}}(t)\}$ is computed, and the EPS $x_{\mathrm{eps}}(t)$ of the sub-band signal $x_{\mathrm{sb}}(t)$ is collected by squaring the absolute FFT values.

Stage 2: Next, based on GDM analysis, an appropriate window is generated to capture the characteristic defect frequencies in the EPS. The GDM-based window size w(j) is defined, as follows:

(5) w(j)={∑i=13exp(−12(α(j−Pi)M2)2)0,Otherwise,Pi−frange≤j≤Pi+frange

Here, j is the index of each frequency bin in the signal spectrum and $P_{i}$ is the $i^{\mathrm{th}}$ harmonic of a bearing defect frequency component (i=3 in this study). In addition, M is the number of frequency bins around each harmonic of BPFO, BPFI, and 2 × BSF, in the specified range of $\left[P_{i}-f_{\mathrm{range}},P_{i}+f_{\mathrm{range}}\right]$, which is calculated as follows:

(6) M=2.frangefresolution

Here, f_range_ defines the frequency range for calculation of the GDM-based window values and f_resolution_ is the frequency resolution (f_resolution_ = 0.2 Hz). Likewise, the parameter α, representing the distribution of Gaussian random variables in Equation (7), is the inverse relation to the standard deviation, which is estimated as follows:

(7) $$\alpha =\frac{M}{N}.\sqrt{-2\ln\beta }$$

Here, β is a fixed constant to ensure the convergence of the window, and is in the range from 0 to 1 (β=0.1 in this paper). The number of considered frequency bins correctly containing the characteristic defect components around their harmonics is denoted by N, as in Stage 2 of Figure 4:

(8) $$\begin{array}{l} N_{\mathrm{outer}}=2\varepsilon \times \mathrm{BPFO}\times \frac{1}{f_{\mathrm{resolution}}}\\ N_{\mathrm{inner}}=\left(n_{\mathrm{sideband}}.F_{r}+\varepsilon .\left(2\mathrm{BPFI}+n_{\mathrm{sideband}}.F_{r}\right)\right)\times \frac{1}{f_{\mathrm{resolution}}}\\ N_{\mathrm{roller}}=\left(n_{\mathrm{sideband}}.\mathrm{FTF}+\varepsilon .\left(4\mathrm{BSF}+n_{\mathrm{sideband}}.\mathrm{FTF}\right)\right)\times \frac{1}{f_{\mathrm{resolution}}} \end{array}$$

Here, the number of considered sidebands appearing around the harmonics of defect components BCI and BCR is determined by $n_{\mathrm{sideband}}=4$. Additionally, $F_{r}$ is the operating frequency or shaft speed in Hz (Fr=speedshaft60). $N_{\mathrm{outer}}$, $N_{\mathrm{inner}}$, and $N_{\mathrm{roller}}$ represent the sizes of frequency bins for outer race, inner race and roller defects. Furthermore, the random variation ε=2% of the defect frequency calculation is also considered to enhance the accuracy when estimating the severity of defects.

Stage 3: Next, the GDM-based window w(j) is multiplied by the initial EPS signal $x_{\mathrm{eps}}(j)$ to capture only the frequency components appearing around the harmonics of the characteristic frequencies of BPFO, BPFI, and 2 × BSF, as shown in Figure 4. This is defined as:

(9) D(j)=xeps(j)×w(j),  1≤j≤fsfresolution

Here, D(j) is the magnitude of the $j^{\mathrm{th}}$ frequency bin in the defect frequency range obtained from the Gaussian window and $f_{s}$ is the sampling rate.

Stage 4: Finally, after identifying the defect frequency components in Step 3, the GDM-based HI for the assessment of a given sub-band is calculated using Equation (11):

(10) $$\begin{array}{l} E_{O}(i)=\sum_{j=1}^{M}O_{i,j}^{2}\\ E_{D}(i)=\sum_{j=1}^{N}D_{i,j}^{2} \end{array}$$

(11) $$\mathrm{HI}=\frac{\sum_{i=1}^{3}E_{D}(i)}{\sum_{i=1}^{3}E_{O}(i)},\;\text{with}\;0\leq \mathrm{HI}\leq 1$$

Here, O_i,j_ is the magnitude of the j^th^ frequency bin around the i^th^ harmonic of the characteristic defect frequency from the initial EPS signal and its corresponding energy E_O_(i). Similarly, D_i,j_ is the amplitude of the j^th^ frequency bin around the i^th^ harmonic of the characteristic defect frequency obtained from Step 3 and its corresponding energy E_D_(i).

In rolling element bearings, the outer race is a fixed component and a defect on the outer race suffers the same impact force both in and outside the load zone. Hence, the EPS of signals for outer race faults has sharp peaks. Defects on the moving parts of bearing, i.e., the inner race and rollers, experience different amounts of forces when they enter and leave the load zone. This results in modulation effects. For inner race faults, the BPFI is modulated by the shaft frequency, as a defect on the inner race enters and leaves the load zone at the same speed as that of the rotating shaft. Whereas, the characteristic frequency of roller faults, i.e., 2 × BSF, is modulated by the cage frequency. Thus, these defects include the sidebands around their fault frequencies, which are the result of amplitude modulation caused by the movement of the defect in and out the load zone. As a result, the EPS for defect components BCI and BCR has a bell shape due to the presence of sidebands in the signal spectra. Therefore, a narrow Gaussian window with frequency range frange=14BPFO is used to evaluate the outer failure, while a large Gaussian window with frequency range frange=12BPFI or BSF is used to evaluate inner and roller failures. After assessing different sub-bands using the GDM-based HI calculation, the optimal sub-band containing explicit information about the bearing defects, which yields the highest HI value, is chosen for reliable bearing fault detection.

## 4. Experimental Results

**Figure 5:**
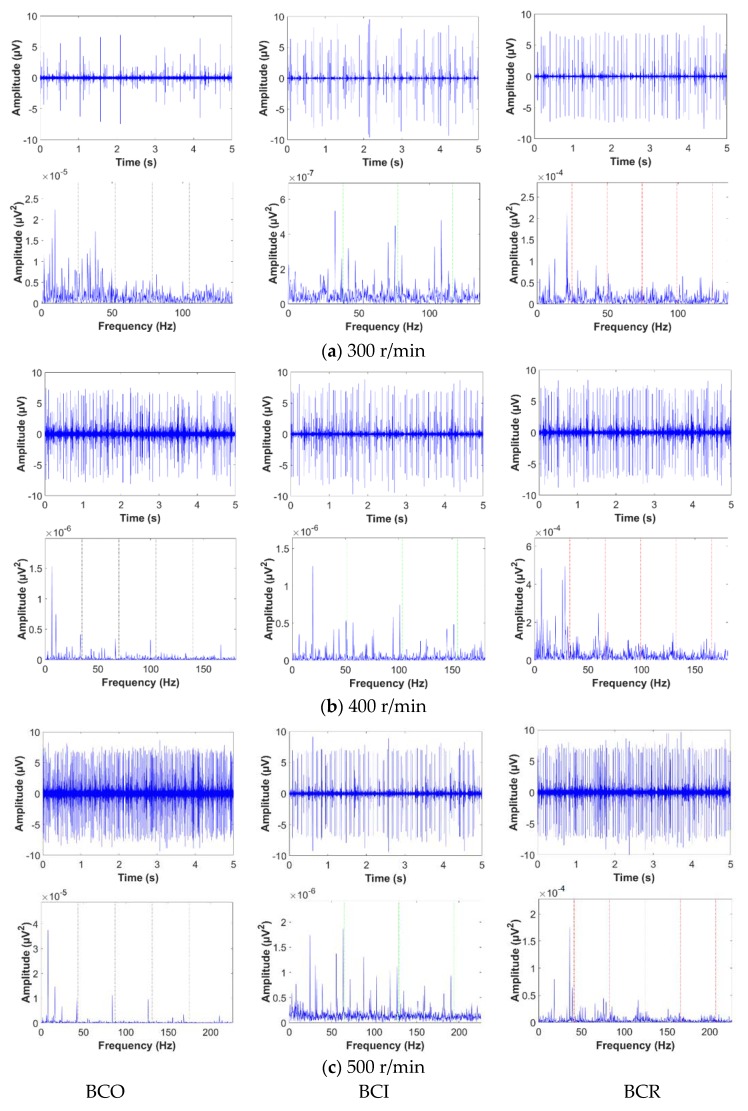
The original acquired AE signals and their initial envelope spectral analysis in frequency domain for bearing conditions related to BCO, BCI, and BCR at the variable rotational speeds: (**a**) 300 r/min, (**b**) 400 r/min, and (**c**) 500 r/min.

**Figure 6:**
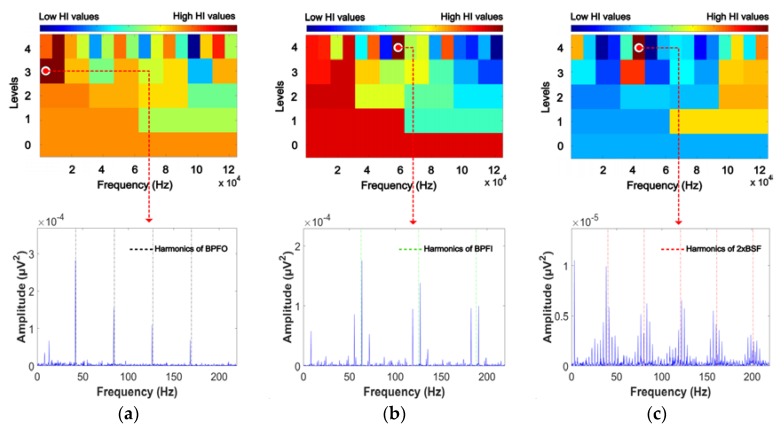
2D visualization analysis for optimal sub-band selection corresponding to the highest HI values of (**a**) BCO, (**b**) BCI, and (**c**) BCR.

This paper presents a comprehensive methodology for early detection of the defect frequency components of BCO, BCI, and BCR based on envelope analysis using a GDM-based window to capture intrinsic information about the failure. The 5 s AE signals of each bearing fault condition are first decomposed into sub-band signals using the DWPT. Then, the bearing defects can be identified by calculating the EPS of $2^{5}-1$ sub-bands from four-level DWPT with the Db4 mother wavelet function. However, abnormal signatures that are characteristic of bearing defects can be present in any sub-band. Figure 5 shows the original time-domain AE signals for different bearing faults at variable rotational speeds and their initial EPS signals. Each fault condition, i.e., outer race, inner race and roller fault, has a unique waveform. Defects on the outer race of a bearing remain stationary, and hence same amount of force is experienced in each rotation, making them less prone to interference by other components. Moreover, if the AE sensor is aligned with the outer race defect and placed directly above it, the acoustic emissions from the outer race defect show the least amount of attenuation since they travel directly to the AE sensor via the smallest path. In contrast, acoustic emissions from roller and inner race defects show more attenuation, as they travel longer distances and are involved in more interference from other components. In Figure 5, the time domain AE signal for BCO condition shows similar attenuation with BCI and BCR. This means that the outer race defect is not aligned with the AE sensor, and hence acoustic emissions from this defect are relatively greater attenuation at low speeds because it travels a longer distance. Based upon the envelope analysis of the raw AE signals, it is difficult to correctly evaluate the defect severity in bearings because the characteristic defect frequencies are not clearly distinguishable in the envelope signal spectra of the raw AE signals. Thus, the signal is decomposed into different sub-bands, and an optimal sub-band is selected using the proposed GDM-based HI for evaluating the EPS to clearly distinguish the different bearing defects. The proposed GDM-based HI is a quantitative metric used in this study for efficient sub-band analysis. As the periodic impulses generated around the harmonics of the defect frequency components of BPFO, BPFI, and 2 × BSF become clearer, the HI value increases. Afterward, the optimal sub-band signal that yields the highest HI value is chosen for extracting the most useful information about the bearing defects. Figure 6 shows the 2D visualization analysis for the HI values obtained from each sub-band, in which three EPS signals related to BCO, BCI, and BCR from the three most informative sub-bands are used for reliable fault detection.

**Figure 7:**
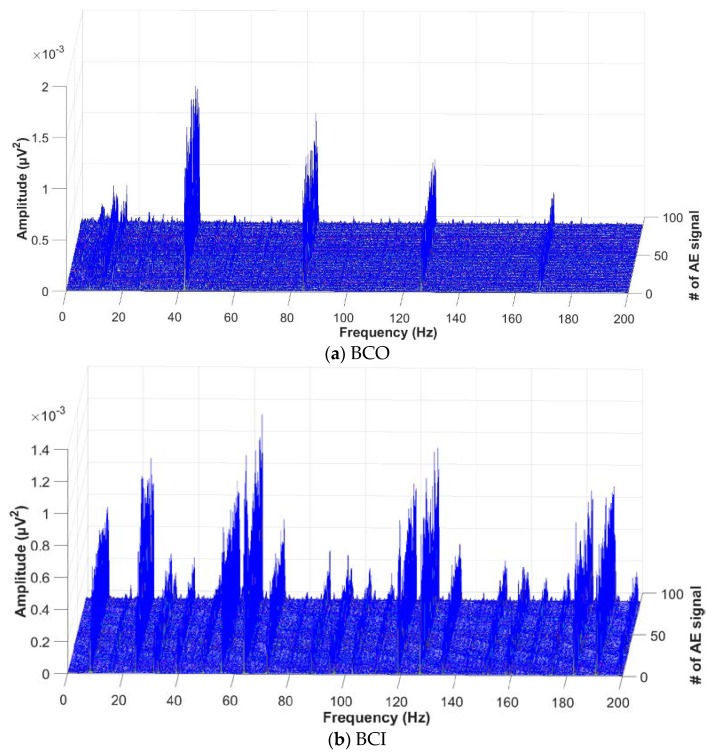

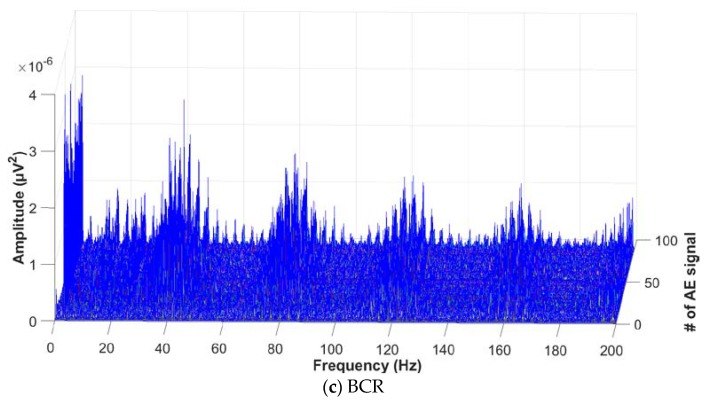
The EPS signals representing the bearing defect conditions of (**a**) BCO, (**b**) BCI, and (**c**) BCR obtained from the optimal sub-bands used for detection.

The performance of the proposed approach is validated through experiments involving the detection of bearing defects caused by cracks on the outer race, inner race, and roller of a bearing. Moreover, these three types of bearing defects are diagnosed under variable rotational speeds of 300 r/min, 400 r/min, and 500 r/min in order to verify the effectiveness of the proposed method in capturing intrinsic information about bearing defects at different speeds. For each defect type, 90 AE signals are acquired at each of the three different rotational speeds. The duration of each AE fault signal is 5 s and is sampled at a frequency of $f_{s}=250\;\mathrm{kHz}$. The optimal sub-band is determined by calculating proposed GDM-based HI for each of the 31 reconstructed sub-band signals. Using the proposed evaluation scheme, the three most informative sub-bands that clearly show the characteristic defect frequencies of BPFO, BPFI, and 2 × BSF around their harmonics are appropriately selected, as shown in Figure 6, by 2D visualization analysis of the calculated HI values based on the envelope signals. Figure 7 illustrates a series of EPS signals for each bearing defect component of BCO, BCI, and BCR, collected from the optimal sub-bands, when the rotation speed is 500 r/min. The abnormal symptoms of bearing defects are accurately identified in the EPS computed from the most informative sub-band. Thus, the proposed GDM-based HI calculation provides an effective metric for measuring periodic impulse protrusion in the EPS, which is precisely proportional to the severity of bearing defects. Accordingly, the proposed approach only captures useful information about failure from the acquired AE signals; this enhances its effectiveness for fault identification in bearings.

The performance of the proposed method in diagnosing different bearing defects i.e., BCO, BCI, and BCR, at different rotational speeds is quantitatively measured using the accuracy (Acc) metric, which is calculated as follows:

(12) $$\mathrm{Acc}=\frac{T_{\mathrm{eps}}}{T_{\mathrm{eps}}+F_{\mathrm{eps}}}\times 100\%$$

Here, T_eps_ is the number of true EPS signals that correctly show characteristic defect frequency components of the bearing, whereas F_eps_ is the number of false EPS signals that do not represent clear signatures of bearing defects. T_eps_ and F_eps_ are determined by observing a series of 90 EPS signals obtained from the optimal sub-bands for each failure condition.

Comparisons of the diagnostic performance of the proposed approach and a state-of-the-art method based on kurtosis are provided in Table 3, Table 4 and Table 5. It can be observed that the kurtosis-based method does give satisfactory performance in identifying bearing defects at 500 r/min. However, its diagnostic performance especially for BCI and BCR is much lower as compared to the performance of the proposed method, especially, at 300 r/min and 400 r/min. This decrease in performance is caused by the inability of kurtosis analysis in identifying the appropriate sub-band for carrying out envelope analysis. This is due to the impact of the harmonics of the operation frequency and noise frequency in the signal spectra. Alternatively, the proposed methodology is suitable for evaluating the severity of bearing defects because it uses GDM-based HI calculations, which are highly efficient in sub-band analysis for correctly detecting the characteristic defect frequencies of bearings. As illustrated in the experimental results, the proposed fault detection approach reaches average accuracies of 95.93%, 98.13%, and 100% at rotational speeds of 300 r/min, 400 r/min, and 500 r/min, respectively.

## 5. Conclusions

This paper presented an effective and reliable fault detection methodology for rolling element bearings based on AE analysis under different rotational speeds. The proposed approach successfully exploited the advantages of DWPT-based signal decomposition and envelope analysis for capturing the characteristic defect frequencies in bearings. The performance of envelope analysis based bearing fault diagnosis hinges on the identification of an appropriate sub-band for carrying out demodulation or calculating the envelope signal. This optimal sub-band clearly shows bearing defect signatures in the resultant EPS. An effective assessment measure was, therefore, proposed for the selection of an optimal sub-band based on GDM-based HI calculation. Consequently, the most informative sub-band, as determined by the highest HI value, was used for calculating the EPS. The resultant EPS was highly effective in highlighting different bearing defect frequencies. The diagnostic performance of the proposed method was compared to a state-of-the-art method based on kurtosis analysis. The results clearly indicated that the proposed method outperformed the conventional approach in terms of detection accuracy for three types of bearing defects, i.e., BCO, BCI, and BCR, at three different rotational speeds.

## Figures and Tables

**Table 1:** Detailed specifications for the AE sensor-based data acquisition.

AE sensor (WSα)	+ Peak sensitivity (V/µbar): −62 dB + Operating frequency range: 100–900 kHz + Resonant frequency: 650 kHz
PCI 2-chanel AE board system	+ ADC: 18-bit 40 MS/s per channel maximum + Frequency response: 1 kHz–3 MHz (at −3 dB points) + Sample rates: 100 KS/s, 200 KS/s, 500 KS/s, 1 MS/s, 2 MS/s, 5 MS/s, 10 MS/s, 20 MS/s, and 40 MS/s are selectable

KS/s: Kilo-Samples per second; MS/s: Mega-Samples per second.

**Table 2:** The magnitudes of characteristic defect frequencies at various speeds.

Crack Size (mm)			Shaft Speed (r/min)	Defect Frequencies (Hz)			
Length	Width	Depth		BPFO	BPFI	2 × BSF	FTF
12	0.49	0.50	300	26.21	38.79	24.87	2.02
			400	34.95	51.72	33.15	2.69
			500	43.68	64.65	41.44	3.36

**Table 3:** The accuracy of bearing defect detection with 300 r/min.

	Proposed			Kurtosis Analysis [22]		
Bearing Defects	BCO	BCI	BCR	BCO	BCI	BCR
$T_{\mathrm{eps}}$	86	90	83	85	87	67
$F_{\mathrm{eps}}$	4	0	7	5	3	23
Acc(%)	95.6	100	92.2	94.4	96.7	74.4

**Table 4:** The accuracy of bearing defect detection with 400 r/min.

	Proposed			Kurtosis Analysis [22]		
Bearing Defects	BCO	BCI	BCR	BCO	BCI	BCR
$T_{\mathrm{eps}}$	90	90	85	87	87	75
$F_{\mathrm{eps}}$	0	0	5	3	3	15
Acc(%)	100	100	94.4	96.7	96.7	83.3

**Table 5:** The accuracy of bearing defect detection with 500 r/min.

	Proposed			Kurtosis Analysis [22]		
Bearing Defects	BCO	BCI	BCR	BCO	BCI	BCR
$T_{\mathrm{eps}}$	90	90	90	90	90	88
$F_{\mathrm{eps}}$	0	0	0	0	0	2
Acc(%)	100	100	100	100	100	97.8
